# Tranexamic acid given into wound reduces postoperative drainage, blood loss, and hospital stay in spinal surgeries: a meta-analysis

**DOI:** 10.1186/s13018-021-02548-6

**Published:** 2021-06-22

**Authors:** Shangyi Hui, Yue Peng, Liyuan Tao, Shengru Wang, Yang Yang, You Du, Jianguo Zhang, Qianyu Zhuang, TARGETS study group

**Affiliations:** 1 Department of Anesthesiology, Peking Union Medical College Hospital, No.1 Shuai Fu Yuan, Wang Fu Jing Street, Beijing, 100730 China; 2 Department of Orthopedics, Peking Union Medical College Hospital, No.1 Shuai Fu Yuan, Wang Fu Jing Street, Beijing, 100730 China; 3 Research Center of Clinical Epidemiology, Peking University 3rd Hospital, Beijing, China

**Keywords:** Topical tranexamic acid, Spinal surgery, Postoperative drainage, Blood loss, Hospital stay

## Abstract

**Background:**

Although intravenous tranexamic acid administration (ivTXA) has prevailed in clinical antifibrinolytic treatment, whether it increases thromboembolic risks has remained controversial. As a potent alternative to ivTXA, topical use of TXA (tTXA) has been successfully applied to attenuate blood loss in various surgical fields while minimizing systemic exposure to TXA. This meta-analysis was conducted to gather scientific evidence for tTXA efficacy on reducing postoperative drainage, blood loss, and the length of hospital stay in spine surgeries.

**Objectives:**

To examine whether topical use of TXA (tTXA) reduces postoperative drainage output and duration, hidden blood loss, hemoglobin level drop, hospital stay, and adverse event rate, we reviewed both randomized and non-randomized controlled trials that assessed the aforementioned efficacies of tTXA compared with placebo in patients undergoing cervical, thoracic, or lumbar spinal surgeries.

**Methods:**

An exhaustive literature search was conducted in MEDLINE and EMBASE databases from January 2000 through March 2020. Measurable outcomes were pooled using Review Manager (RevMan) version 5.0 in a meta-analysis.

**Results:**

Significantly reduced postoperative drainage output (weighted mean difference [WMD]= − 160.62 ml, 95% confidence interval (95% CI) [− 203.41, − 117.83]; p < .00001) and duration (WMD= − 0.75 days, 95% CI [− 1.09, − 0.40]; p < .0001), perioperative hidden blood loss (WMD= − 91.18ml, 95% CI [− 121.42, − 60.94]; p < .00001), and length of hospital stay (WMD= − 1.32 days, 95% CI [− 1.90, − 0.74]; p < .00001) were observed in tTXA group. Pooled effect for Hb level drop with tTXA vs placebo crossed the equivalent line by a mere 0.05 g/dL, with the predominant distribution of 95% confidence interval (CI) favoring tTXA use.

**Conclusions:**

With the most comprehensive literature inclusion up to the present, this meta-analysis suggests that tTXA use in spinal surgeries significantly reduces postoperative drainage, hidden blood loss, and hospital stay duration. The pooled effect also suggests that tTXA appears more effective than placebo in preserving postoperative Hb level, which needs further validation by future studies.

## Background

Spinal surgeries are commonly associated with massive perioperative blood losses, both visible in the surgical field and hidden into dead space, which leads to excessive fibrinolysis within the wound as a result of acute consumptive coagulopathy [1]. As a well-accepted antifibrinolytic agent, tranexamic acid (TXA) has been conventionally administered through the intravenous route and has achieved satisfactory outcomes in minimizing total blood loss [2–4]. However, accumulating evidence has questioned the safety of intravenous use of TXA (ivTXA), as the treatment has been reported to cause postoperative seizures and systemic thrombogenicity [5, 6]. A 3% incidence rate of thromboembolic events has been reported after high-dose ivTXA during spinal fusion surgery [7], while ivTXA dose ≥ 100 mg/kg has been identified as a risk factor for developing postoperative TXA-related seizures and strokes [8].

As a potent alternative to ivTXA, topical TXA (tTXA) inhibits the fibrinolytic process in situ while minimizing systemic exposure to ivTXA. tTXA treatment has been successfully applied to attenuate visible and hidden blood losses in hip and knee arthroplasty [9–12]. Sporadic randomized and non-randomized controlled trials regarding tTXA use have been recently reported in spinal surgeries. However, high-quality evidence from strictly performed meta-analyses on tTXA efficacy and safety is still lacking due to the limited scope of literature inclusion and underpowered analysis [13, 14].

Therefore, we conducted this meta-analysis with all available research to gather scientific evidence for the differences between tTXA versus placebo in postoperative drainage, hidden blood loss, hemoglobin level drop, hospital stay, and adverse event rate. To the best of our knowledge, this study was conducted with the most comprehensive study inclusion up to the present, and we have followed Preferred Reporting Items for Systematic Reviews and Meta-Analyses guidelines to help improve the reporting quality of our study.

## Materials and methods

### Literature search

To identify the published articles on spine surgery and tTXA delivery, an exhaustive literature search of EMBASE and MEDLINE, both manual and computer-assisted, was conducted according to the predetermined search strategies (Appendix 1; Appendix 2). Registered clinical trials on the use of tTXA in spinal surgeries were searched on the same day, using the US National Institutes of Health database (www.ClinicalTrials.gov), the World Health Organization International Clinical Trials Registry Platform (http://www.who.int/ictrp/en/), and the International Standard Randomized Controlled Trial Number ISRCTN registry (https://www.isrctn.com). The following search terms were used in all databases: (1) local/topical tranexamic acid (including all relevant synonyms/usages, i.e., topical TXA, tTXA, local TXA); AND (2) spine surgery OR spinal surgery. Prospective randomized controlled trials (RCTs), non-RCT studies including quasi-randomized trials (qi-RCTs), retrospective cohort studies, and case-control studies that compared tTXA infiltration with no tTXA administration at wound closure in spinal surgeries were distinguished from January 2000 through March 2020. There was no restriction on the reporting language of the article. Reference lists in studies, reviews, and previous meta-analyses were checked to identify any initially omitted studies. Particular attention was paid to duplicate reports; when studies were published as an abstract and an original article, only the latter was considered.

### Study selection

To be selected, studies should meet the following inclusion criteria: (1) patients underwent cervical, thoracic, or lumbar spinal surgeries irrespective of the anterior or posterior approach; (2) topical administration of TXA (tTXA) was compared with no tTXA administration at wound closure; and (3) studies have evaluated the efficacy or safety of tTXA using at least one of the following endpoints: (a) output and duration of postoperative drainage, (b) hidden blood loss (HBL), (c) hemoglobin (Hb) level changes from baseline, (d) hospital stay, or (e) numbers of postoperative adverse events, including wound infections and thrombosis events. Once the studies met the eligible criteria, they would be included even published in gray literature.

### Methodology quality assessment

Two reviewers independently scanned the quality of the eligible studies; a third reviewer would solve discrepancies. We assessed the study quality using the Newcastle-Ottawa Scale described by Wells et al. [15], in which a study was judged on three broad perspectives: selection of the study groups, comparability of the groups, and ascertainment of either the exposure or outcome of interest. Studies with 7 points or higher were considered high-quality research and were included in the meta-analysis.

### Risk of bias assessment

Two reviewers independently assessed the risk of bias of the enrolled studies. We assessed the risk of bias for randomized trials using the criteria specified in the Cochrane Handbook for Systematic Reviews of Interventions [16]. For non-randomized studies, we used the ROBINS-I tool criteria for assessing the risk of bias [17]. A third reviewer solved discrepancies between the reviewers.

### Data extraction

Predefined data from individual trials were extracted independently by two authors. The data extracted included both study characteristics and measuring outcomes. The name of the first author, country, year of publication, design type, tTXA delivery methods, surgical procedure, and quality assessment were recorded as study characteristics, whereas output and duration of postoperative drainage, postoperative Hb level change, perioperative hidden blood loss, length of hospital stay, and the number of postoperative adverse events were extracted as the measuring outcomes.

### Statistical analysis

Statistical analyses were conducted using Review Manager (RevMan) version 5.3 (The Cochrane Library, Oxford, UK). The mean and standard deviations were pooled to a mean difference (MD) and 95% confidence interval (CI) for continuous outcomes. When median and interquartile range (IQR) were reported rather than mean and SD, the data were converted to the desired format according to the Cochrane Handbook for Systematic Reviews of Interventions [16]. Computation of Hb change from baseline and combination of subgroup measurements were also conducted following the Handbook's instructions [16]. Odds ratios (OR) and 95% confidence interval (CI) were calculated for dichotomous outcomes.

The quantity of heterogeneity was assessed using I^2^ statistics. When there was no statistical evidence of substantial heterogeneity (I^2^ ≤ 50%), a fixed-effect model was adopted; otherwise, a random-effect model was chosen.

### Strength of evidence assessment

Two reviewers independently assessed the required 5 domains (study limitations, consistency, directness, precision, and reporting bias) for each major outcome, with discrepancies solved by a third reviewer. The overall strength of evidence (SOE) was then established by incorporating the 5 domains into an overall grade, which was denoted as high, moderate, low, or insufficient. The SOE assessment procedures were strictly conducted according to the series paper by the US Agency for Healthcare Research and Quality (AHRQ) and the Evidence-based Practice Center (EPC) program [18] and presented as per the suggested approach in the EPC update [19].

## Results

### Literature search and study selection

**Fig. 1:**
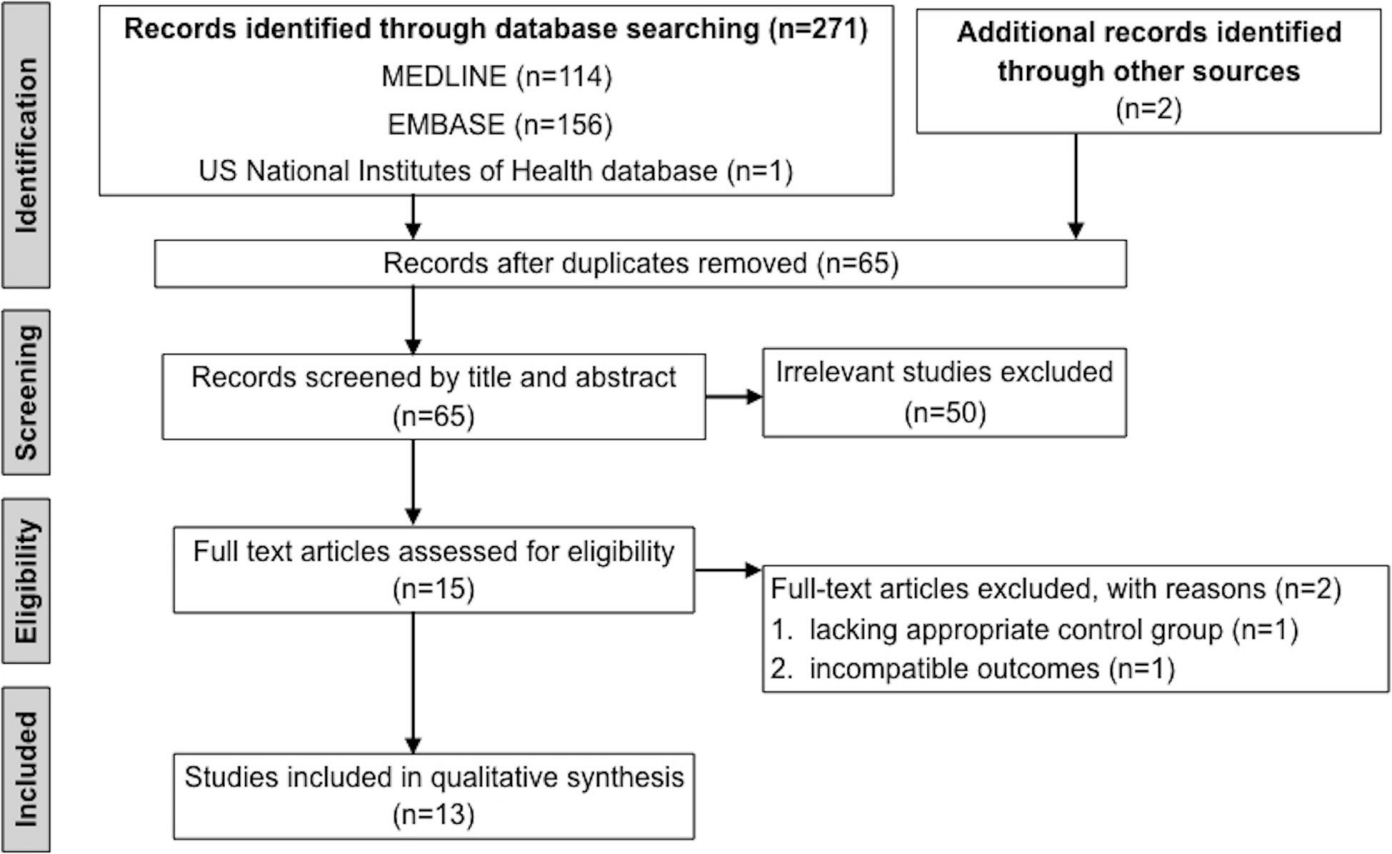
Study flow chart. Study selection process in this meta-analysis

Figure 1 shows the flow chart for identifying eligible studies. The search strategy yielded a total of 270 articles and 1 registered clinical trial from the electronic databases (MEDLINE = 114, EMBASE = 156, US National Institutes of Health database = 1). Two studies were identified through checking the reference lists in previous reviews and meta-analyses. After excluding 208 studies as literature appearing in more than 1 database, 65 studies remained. After screening by titles and abstracts, 15 studies remained potentially relevant. After full-text review, 2 studies were excluded because of the lack of an appropriate control group with either a placebo or no tTXA, or outcome measures that were not specified in the meta-analysis. Finally, 9 RCTs [20–28] and 4 non-RCT studies [29–32] involving 882 patients were included in the final analysis, with individual sample sizes ranging from 29 to 100 patients. Among them, 11 studies were published in English, 1 study in Chinese, and 1 study in Persian. The included studies were determined as above 7 points according to the 9-star Newcastle-Ottawa Scale. The characteristics of included studies are presented in Table 1.

**Table 1:** Characteristics of studies included

Ref.	First Author	Country	Year	Size(E/C)	Design	Procedures	tTXA Regimen	Quality^a^
20	Claus	Norway	2003	16/14	RCT	Operations for low back pain	500 mg TXA added into 50 ml 0.9% NS	7
21	Hoshang	Iran	2010	50/50	RCT	One/two level laminectomy	250 mg TXA added into 5 ml 0.9% NS	8
22	Liang	China	2016	30/30	RCT	Multilevel posterior lumbar degenerative procedures	2000 mg TXA (in 20 ml 0.9% NS) soaked Gelfoam (100 cm^2^)	7
29	Ren	China	2017	50/50	nRCT	Posterior lumbar spinal fusion surgery	1 g TXA added into 100 ml 0.9% NS	7
30	Zhinan	China	2017	50/50	nRCT	Posterior lumbar spinal fusion surgery	1 g TXA added into 100 ml 0.9% NS	7
23	Xu	China	2017	40/40	RCT	Total laminectomy with pedicle screw instrumentation	1 g TXA added into 100 ml 0.9% NS	8
24	Mu	China	2019	39/42	RCT	Posterior lumbar interbody fusion	1 g TXA (in 50 ml 0.9% NS) soaked gelatin sponge	7
25	Wood	USA	2018	12/17	RCT	Spinal surgery	3 g TXA irrigated in the wound prior to closure	7
31	Liang	China	2020	20/20	nRCT	Posterior lumbar decompression and fusion	1 ampoule (10% transamin, 10 ml, 1000 mg TXA; Daiichi-Sankyo, Tokyo, Japan)	7
26	Weera	Thailand	2019	29/28	RCT	Long-segment instrumented fusion without decompression	1 g TXA added into 20 ml 0.9% NS	7
27	Xu	China	2020	30/30	RCT	Posterior lumbar interbody fusion	1 g TXA added into 100 ml 0.9% NS	7
32	Weera	Thailand	2018	35/38	nRCT	Long-segment instrumented fusion without decompression	1 g TXA added into 20 ml 0.9% NS	7
28	Han	China	2019	48/24	RCT	Multilevel thoracolumbar bone graft fusion and internal fixation	500 mg/1000 mg TXA added into 100 ml 0.9% NS	7

*Ref.*, reference; *E/C*, experimental group/control group; *RCT*, randomized control trial; *nRCT*, non-randomized control trial; *tXA*, topical use of tranexamic acid. ^a^ Study quality assessed using the Newcastle-Ottawa Scale (NOS)

**Fig. 2:**
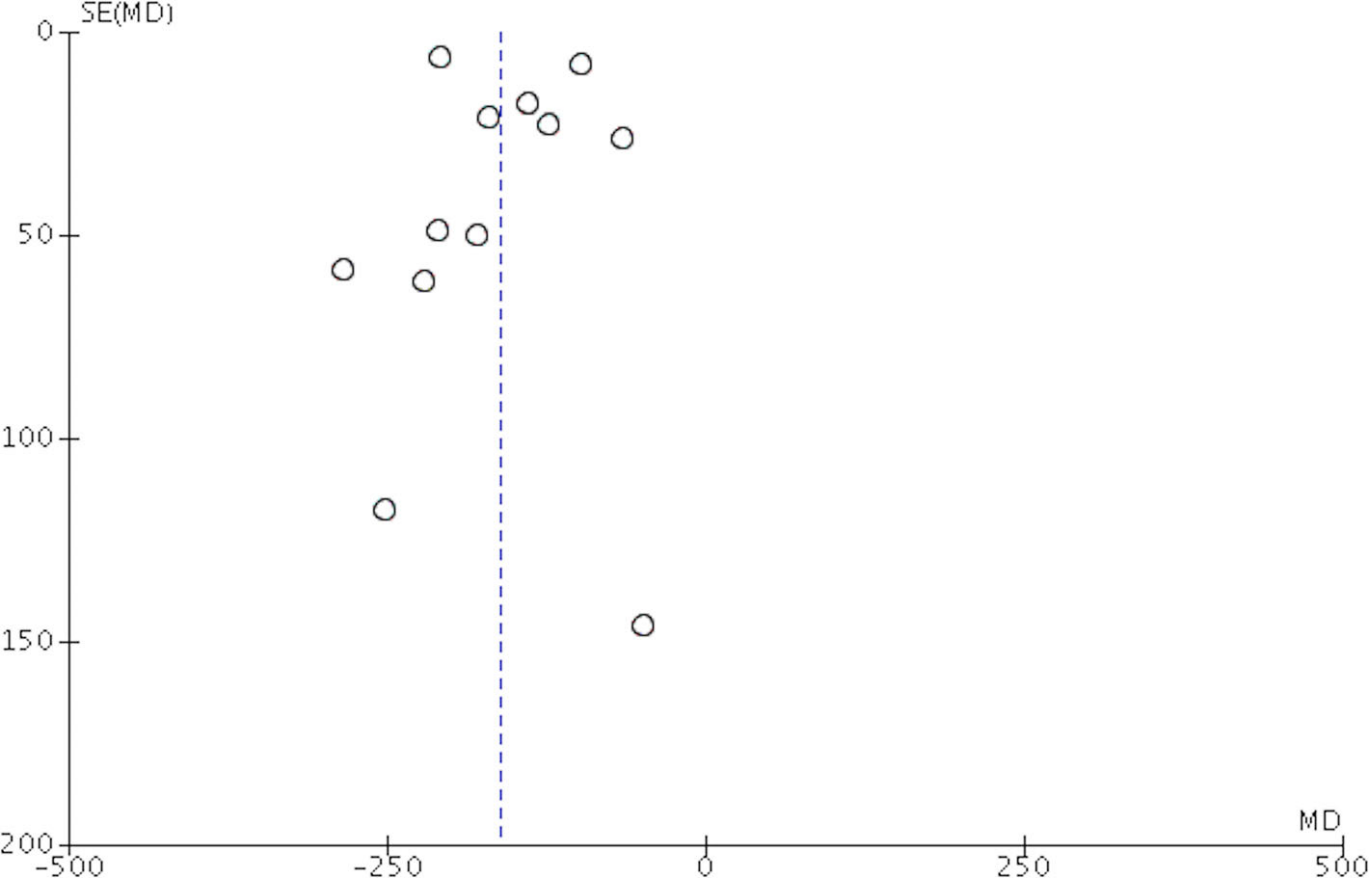
Funnel plot of postoperative drainage output. Funnel plot of tTXA use was compared with placebo on the postoperative drainage output. tTXA, topical use of tranexamic acid

Postoperative drainage output was used to generate the funnel plot analysis of publication bias (Fig. 2). The asymmetric characteristic of the resultant plot indicated the presence of publication bias.

### Risk of bias in included studies

**Fig. 3:**
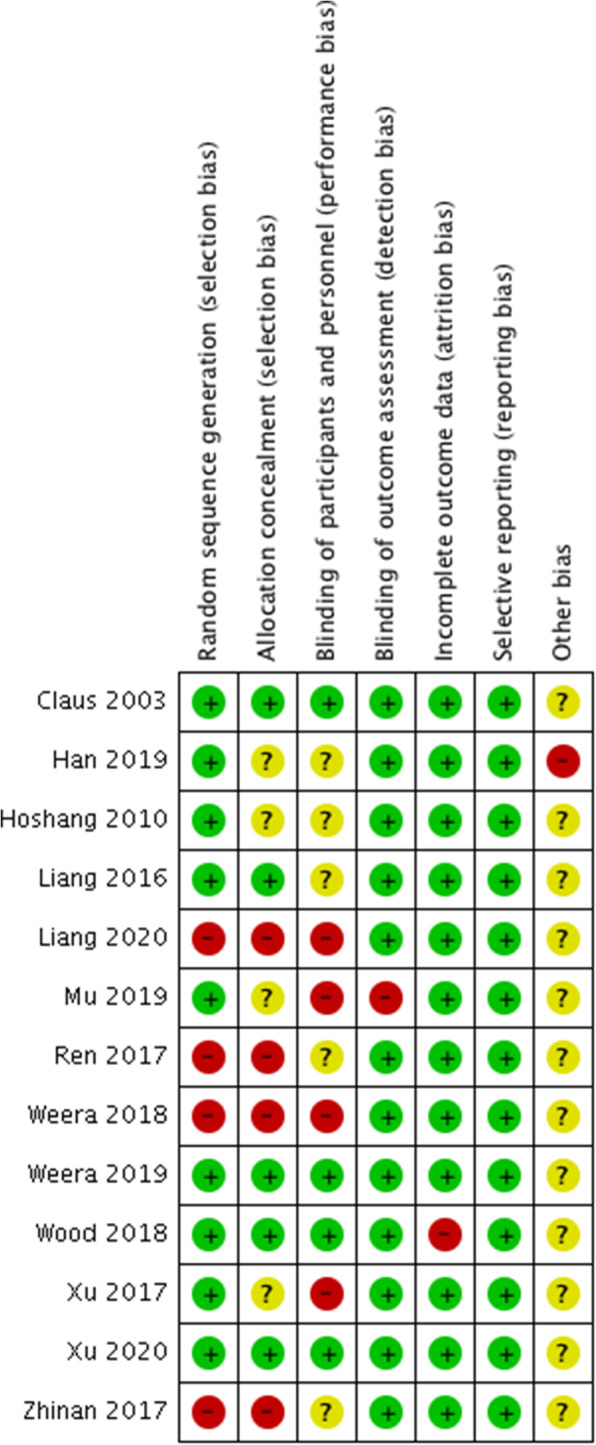
Risk of bias summary: review authors’ judgments about each risk of bias item for each included study

**Fig. 4:**
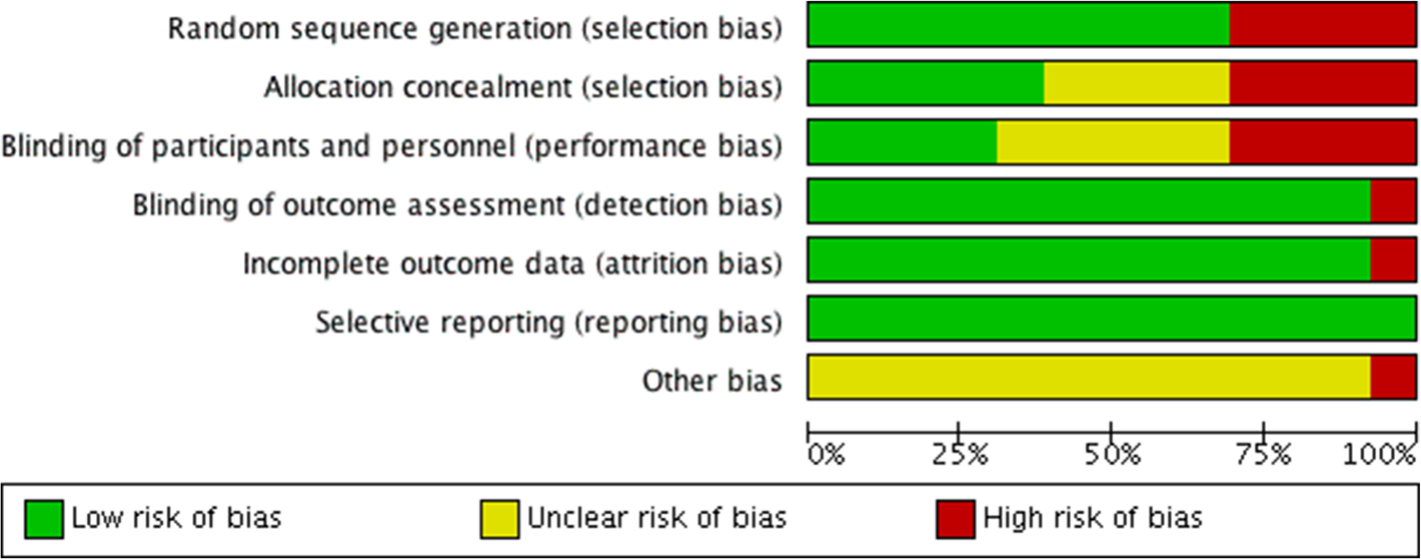
Risk of bias graph: review authors’ judgments about each risk of bias item presented as percentages across all included studies

The risk of bias in included studies was summarized in Figs. 3 and 4. All randomized studies were at low risk of selection bias, with the randomization and allocation process being clearly reported. All non-randomized studies were at a high risk of selection bias, as patients in Ren (2017), Zhinan (2017), Liang (2020), and Weera (2018) [29–32] were most likely selected for different study treatments based on clinical factors. The blinding process was not followed in 1 randomized study [24] due to some patients requesting more information to understand their diagnosis and treatment fully.

### Postoperative drainage output

**Fig. 5:**
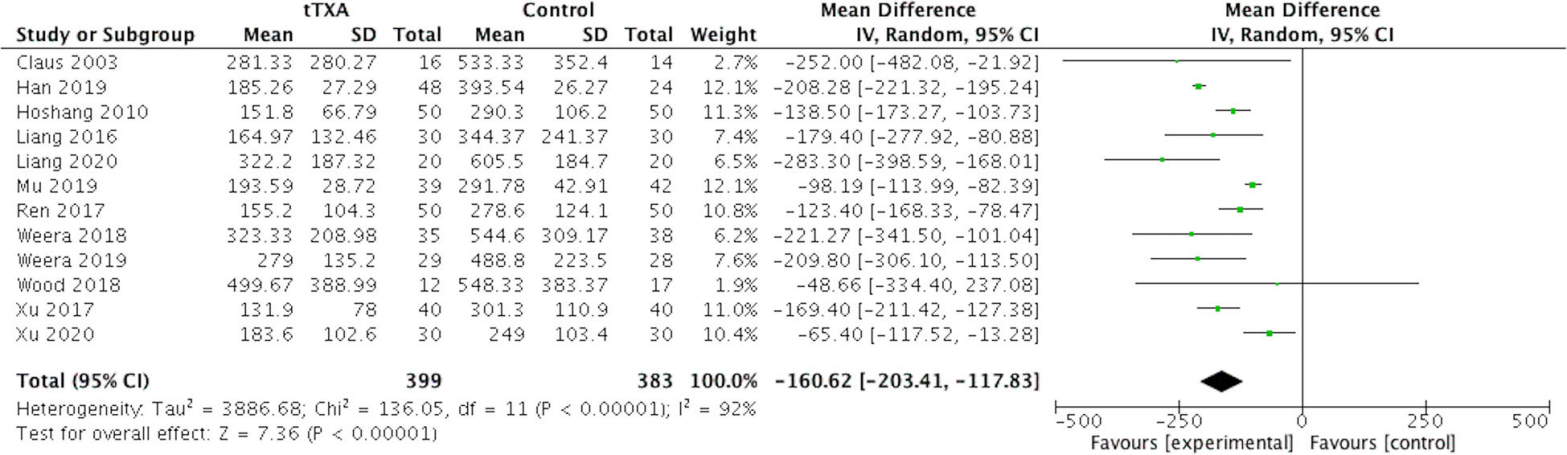
Forest plot of postoperative drainage output. CI, confidence interval; IV, inverse variance methods; tTXA, topical use of tranexamic acid; Random, random-effects modeling

Twelve studies (n=782) were included in the meta-analysis for postoperative drainage output. Pooled results indicated that tTXA application was more effective than the placebo in reducing postoperative drainage (weighted mean difference [WMD]= − 160.62 ml, 95% confidence interval (95% CI) [− 203.41, − 117.83]; p< .00001) (Fig. 5).

### Postoperative drainage duration

**Fig. 6:**
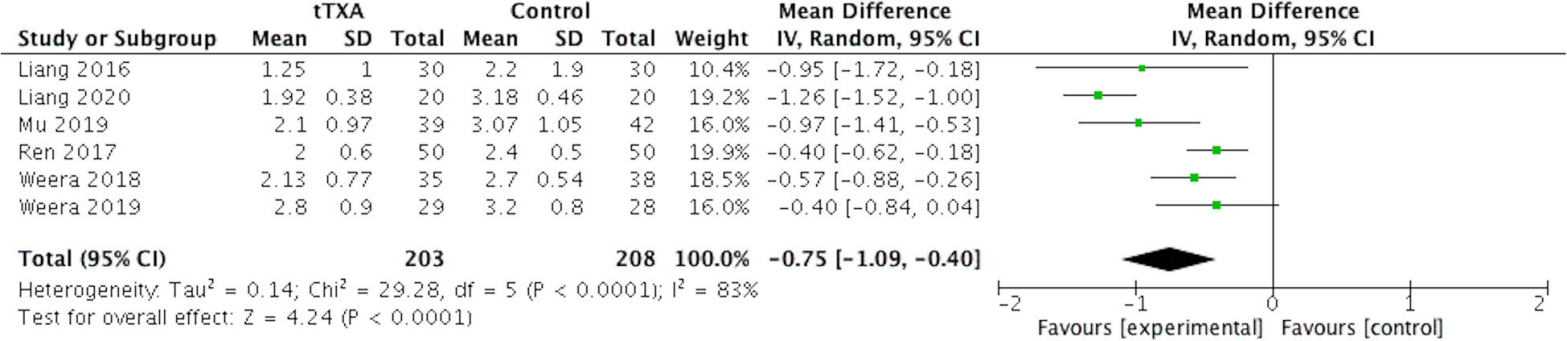
Forest plot of postoperative drainage duration. CI, confidence interval; IV, inverse variance methods; tTXA, topical use of tranexamic acid; Random, random-effects modeling

A total of 6 studies (n=411) provided data on drainage retention duration. Pooled results indicated that tTXA application was more effective than placebo in reducing the duration of postoperative drainage (WMD= − 0.75 days, 95% CI [− 1.09, − 0.40]; p< .0001) (Fig. 6).

### Hidden blood loss (HBL)

**Fig. 7:**
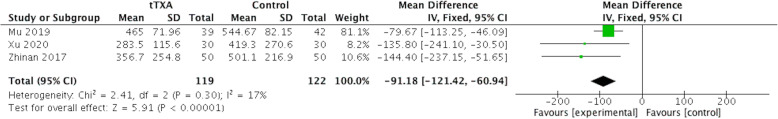
Forest plot of perioperative hidden blood loss. CI, confidence interval; IV, inverse variance methods; tTXA, topical use of tranexamic acid; Fixed, fixed-effects modeling

Three studies (n=241) were included in the meta-analysis for perioperative HBL. Pooled results revealed a significant reduction in HBL with the application of tTXA compared with placebo (WMD= − 91.18 ml, 95% CI [− 121.42, − 60.94]; p< .00001) (Fig. 7).

### Hemoglobin (Hb) level drop

**Fig. 8:**
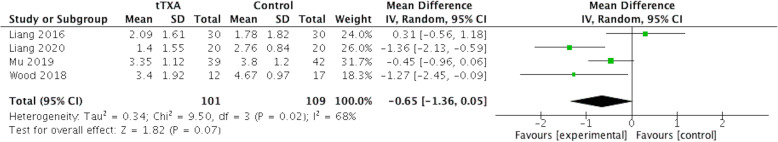
Forest plot of postoperative hemoglobin (Hb) level drop. CI, confidence interval; IV, inverse variance methods; tTXA, topical use of tranexamic acid; Random, random-effects modeling

Data were extracted from 4 studies (n=210) for the meta-analysis of postoperative Hb level drop. The pooled 95% CI resided predominantly on the left side of the equivalent line and crossed the line by a mere 0.05 g/dL, indicating that tTXA application caused less Hb drop compared with the placebo in most cases (WMD= − 0.65 g/dL, 95% CI [− 1.36, 0.05]; p=0.07) (Fig. 8).

### Length of hospital stay (LOH)

**Fig. 9:**
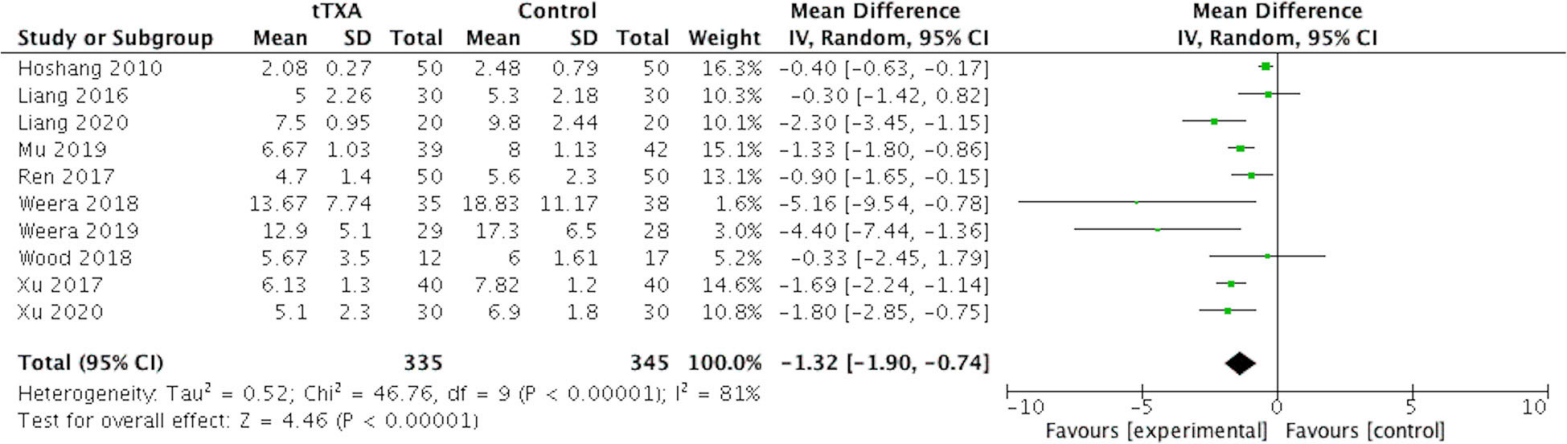
Forest plot of length of hospital stay (LOH). CI, confidence interval; IV, inverse variance methods; tTXA, topical use of tranexamic acid; Random, random-effects modeling

Data on hospital stay were available in 10 studies with 680 patients. Pooled results indicated a statistically significant reduction in LOH with tTXA treatment compared with placebo (WMD= − 1.32 days, 95% CI [− 1.90, − 0.74]; p< .00001) (Fig. 9).

### Adverse event (AE) rate

**Fig. 10:**
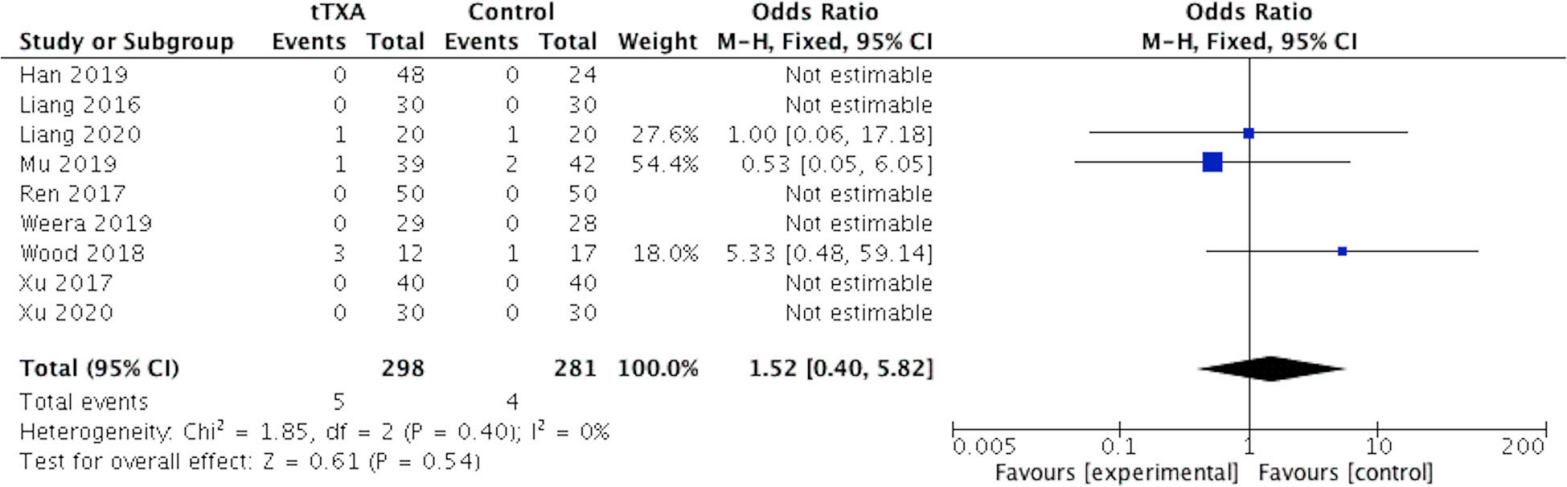
Forest plot of adverse event rate. CI, confidence interval; M-H, Mantel-Haenszel; tTXA, topical use of tranexamic acid; Fixed, fixed-effects modeling

This meta-analysis did not show a significantly increased risk for postoperative adverse events with tTXA treatment. Nine articles (n=579) were included in the meta-analysis. Nine adverse events were reported in the included studies, with 1 case of myocardial infarction and 4 cases of wound infection in the tTXA group, and 4 cases of wound infection in the placebo group. The pooled result indicated event rates of 5 of 298 versus 4 of 281 for tTXA administration compared with placebo treatment, with OR 1.52 (0.40, 5.82), p = .54 (Fig. 10).

### Strength of evidence (SOE)

Outcomes were categorized into major outcomes, patient-centered outcomes, and adverse events. The strength of evidence (SOE) for each outcome was illustrated in Table 2. Except for hemoglobin (Hb) level drop, all other major outcomes were determined to be of moderate-high evidence quality. SOE for Hb level drop was denoted as insufficient, since no conclusion can be drawn from the existing findings. The patient-centered outcome was denoted as having moderate SOE, with consistent and precise effect yielded from medium-risk studies. However, with sparse events reported in small-sized studies, SOE for adverse events was denoted as insufficient in this study.

**Table 2:** Summary of key outcomes, findings, and strength of evidence. Abbreviations: RCT, randomized controlled trial; *SMD*, standardized mean difference; *CI*, confidence interval; *TXA*, tranexamic acid; *tTXA*, topical use of TXA

Outcome	Study design: No. studies (***N***)	Findings and direction (magnitude) of effect	Strength of evidence
**Major outcomes**			
Postoperative drainage output	RCT: 9 (569) Non-RCT: 3 (213)	RCTs with sufficient sample sizes and non-RCTs with low risks of study limitations found a consistent effect that tTXA reduced postoperative drainage output more than placebo, with overall SMD − 160.62 (− 302.41, − 117.83).	High
Postoperative drainage duration	RCT: 3 (198) Non-RCT: 3 (213)	RCTs and non-RCTs with medium-level study limitations found a consistent and precise effect that tTXA reduced postoperative drainage duration more than placebo, with overall SMD − 0.75 (− 1.09, − 0.40).	Moderate
Hidden blood loss	RCT: 2 (141) Non-RCT: 1 (100)	Consistent and precise effect was found in studies with medium-level study limitations that tTXA reduced perioperative hidden blood loss more than placebo by SMD − 91.18 (− 121.42, − 60.94).	Moderate
Hemoglobin level drop	RCT: 3 (179) Non-RCT: 1 (40)	Few studies included. Direction of effect in non-RCT conflicted with that in RCTs, yielding an overall SMD − 0.65 (− 1.36, 0.05) with a wide (imprecise) CI.	Insufficient
**Other patient-centered outcomes**			
Length of hospital stay (LOH)	RCT: 7 (467) Non-RCT: 3 (213)	Studies with medium-level study limitations yielded consistent and precise effect that tTXA reduced LOH more than placebo by SMD − 1.32 (− 1.90, − 0.74).	Moderate
**Adverse events**			
Rod fracture	Non-RCT: 1(40)	Only a single event was reported in one small non-RCT.	Insufficient
Wound infection	RCT: 2 (110) Non-RCT: 1(40)	Three events were reported in a few small RCT and non-RCTs, finding no significant difference in wound infection rate.	Insufficient
Myocardial infarction	RCT: 1 (29)	Only a single event was reported in one small RCT.	Insufficient

## Discussion

Topical application of TXA was first reported to effectively reduce blood loss in spine surgery in 2003 [20]. The researchers also reported significantly lower concentrations of plasmin/α_2_-antiplasmin (PAP) and D-dimer in drained blood by tTXA treatment compared with placebo, indicating that tTXA contributes to inhibiting blood loss by preventing excessive fibrinolysis. The suppression of fibrinolytic activity by tTXA has spurred an investigation into its use in spinal surgeries. With the most comprehensive inclusion of qualified studies, this study aimed to pool all the current evidence for the efficacy of tTXA in reducing postoperative drainage, attenuating HBL, shortening hospital stay, and the safety of its use in spinal surgeries by meta-analysis.

Our results revealed that tTXA application led to a significant reduction in postoperative drainage output and duration. Prolonged postoperative drainage may aggravate wound contamination and compromise postoperative rehabilitation, which poses a great challenge for patient recovery after surgery. To alleviate such concerns, a team of spine surgeons have conducted consecutive clinical trials, including a retrospective study in 2018 [32] and a prospective study in 2019 [26]. Both studies reached consistent conclusions that tTXA infiltration on decorticated laminae surface could significantly reduce postoperative drainage. With enriched evidence from the largest number of included studies so far, our analysis further validated that TXA use effectively reduced both the output and duration of postoperative drainage in spinal surgeries.

While the visible postoperative drainage has gained extensive research interests, the invisible hidden blood loss has long been overlooked, largely due to its residual in dead spaces and extravasation into tissues. The concept of HBL was first put forth in 2000 [33]. According to previous studies, both ours and others’, quantities of HBL in spine surgeries are substantial. Smorgick et al. reported that HBL accounted for 39–42% of TBL in primary/revision posterior spinal fusion surgeries [34], and a retrospective study conducted at our center reported the percentage as high as 47% [35]. HBL has also been reported as significantly associated with increased postoperative complications and length of hospital stay (LOH) [36]. Therefore, we preliminarily investigated the efficacy of tTXA application on HBL management in this meta-analysis. According to our pooled results, the application of tTXA at wound closure significantly reduced HBL in spinal surgeries. The underlying mechanism may be that tTXA directly blocked the lysine binding sites of plasminogen and retarded fibrinolysis, thereby stabilizing the blood clot and reducing HBL within the wound. However, studies regarding HBL management by tTXA application have been scarce. This meta-analysis has included all the relevant publications and provided essential evidence for the positive effect of tTXA on HBL control. Larger scale randomized controlled trials are still required to further investigate the effect of tTXA application as a potent measure of reducing perioperative HBL.

It is reasonable to predict that decreased visible drainage and invisible HBL amounts should collaboratively lead to lesser Hb level drop. In our pooled analysis, we observed an obvious tendency of tTXA as more efficient than placebo in preserving Hb level, despite the fact that the 95% CI of Hb change crossed the equivalent line by a mere 0.05 g/dL. This could be due to the fact that data sources of Hb level changes were different from those of postoperative drainage and HBL. Considering the predominant distribution of the interval favoring tTXA use, we deduced that the statistical insignificance should not impede the clinical significance of tTXA application in conserving blood. With existing data from only four included studies [22, 24, 25, 31], further well-designed clinical trials are expected to provide more validated evidence over this issue.

Our pooled results also indicated that tTXA use significantly decreased the length of hospital stay (LOH). Reasons for the early discharge from the hospital are multiple, including shortened drainage maintenance, less postoperative bleeding, lower incidence of anemia, and the resulting better conditions that lead to earlier functional exercises. Such improvements suggest that tTXA treatment not only optimizes the enhanced recovery after surgery (ERAS) scheme in spinal surgeries, but also benefits reducing hospital costs [37, 38]. Yet, it should be noted that since the included trials were not designed with LOH as the primary outcome, more powerful evidence from well-designed trials is still needed in the future to confirm the efficacy of tTXA in reducing hospital stay.

It should also be noted that our pooled analysis revealed no statistically significant differences for adverse event rates between the two groups. tTXA use provided a maximal concentration of TXA at the bleeding site with minimal systemic exposure of TXA, and therefore attenuated the potential risks for thromboembolic complication and neurotoxicity [39, 40]. Additionally, strict sterilization was followed for tTXA delivery in each trial, which has constrained the risks of wound complications. However, it could also be due to the fact that the studies included in our analysis were designed to assess efficacy rather than adverse events. Therefore, great care should still be taken regarding the safety profile of tTXA, and more studies specifically screening for tTXA-related adverse events are still expected.

Our study has several substantial strengths compared with the existing meta-analyses regarding tTXA use in spinal surgeries [13, 14]. Firstly, this meta-analysis was conducted with the most comprehensive study inclusion up to the present. Secondly, postoperative drainage duration and hidden blood loss were pooled as measuring outcomes for the first time. Thirdly, this study has shed light on the combined use of intravenous TXA administration (ivTXA) and tTXA application in spinal surgeries. Our previous study has confirmed the efficacy of ivTXA in reducing perioperative blood losses, leaving its safety undetermined [41]. Combination use of both TXA regimens may reduce ivTXA dosages while benefiting local hemostasis, thereby attenuating potential risks of thromboembolic events. To address this issue, tTXA and ivTXA applications are compared in our ongoing prospective TARGETS trials, with further evidence for combining tTXA to ivTXA use in spinal surgeries to be expected [42].

However, this study has several limitations that need to be addressed. Different topical routes were adopted in the included studies, including wound irrigation at the conclusion of operation, applying TXA-soaked sponges, or injection via drainage plus drain-clamping. Varied delivery methods may introduce heterogeneity into pooled analyses. Furthermore, the enrolled ages, diagnoses, surgical procedures, fusion levels, and TXA dosages varied from one study to another, which may also introduce considerable bias in the analyses.

## Conclusions

With the most comprehensive literature inclusion up to the present, this meta-analysis suggests that tTXA use in spinal surgeries significantly reduces postoperative drainage, hidden blood loss, and hospital stay duration. The pooled effect also suggests that tTXA appears more effective than placebo in preserving postoperative Hb level, which needs further validation by future studies. However, considerable biases may be introduced due to the diagnoses, surgical procedures, fusion levels, tTXA delivery routes, and dosages varying from one study to another. Therefore, larger prospective trials with specifically designed outcomes are still required to define the optimal delivery method and to confirm the safety of tTXA use in spinal surgeries.

## Data Availability

The datasets used and/or analyzed during the current study are available from the corresponding author on reasonable request.

## Appendix 1. MEDLINE Search Strategy

1. exp Antifibrinolytic Agents/
2. (anti-fibrinolytic* or antifibrinolytic* or antifibrinolysin* or anti-fibrinolysin* or antiplasmin* or anti-plasmin* or ((plasmin or fibrinolysis) adj3 inhibitor*)).ab,ti.
3. exp Tranexamic Acid/
4. (tranexamic or Cyclohexanecarboxylic Acid* or Methylamine* or trans-4-aminomethyl-cyclohexanecarboxylic acid* or t-amcha or amca or kabi 2161 or transamin* or exacyl or amchafibrin or spotof or cyklokapron or ugurol oramino methylcyclohexane carboxylate or aminomethylcyclohexanecarbonic acid or aminomethylcyclohexanecarboxylic acid or AMCHA or amchafibrin or amikapron or aminomethyl cyclohexane carboxylic acid or aminomethyl cyclohexanecarboxylic acid or aminomethylcyclohexane carbonic acid or aminomethylcyclohexane carboxylic acid or aminomethylcyclohexanecarbonic acid or aminomethylcyclohexanecarboxylic acid or aminomethylcyclohexanocarboxylic acid or aminomethylcyclohexanoic acid or amstat or anvito or cl?65336 or cl65336 or cyclocapron or cyclokapron or cyklocapron or exacyl or frenolyse or hexacapron or hexakapron or tranex or TXA).ab,ti.
5. 1 or 2 or 3 or 4
6. exp Administration, Topical/
7. (topical* or (local* adj3 appl*) or irrigat* or spray*).ab,ti.
8. 6 or 7
9. 5 and 8
10. exp Spine/
11. exp Spinal Fusion/
12. 10 or 11
13. 9 and 12
14. randomized controlled trial.pt.
15. controlled clinical trial.pt.
16. randomized.ab.
17. placebo.ab.
18. clinical trials as topic.sh.
19. randomly.ab.
20. trial.ti.
21. exp Cohort Studies/
22. (cohort* or prospective* or retrospective*).mp.
23. 14 or 15 or 16 or 17 or 18 or 19 or 20 or 21 or 22
24. exp animals/ not humans.sh.
25. 23 not 24
26. 13 and 25

## Appendix 2. EMBASE Search Strategy

1. exp Antifibrinolytic Agents/

2. (anti-fibrinolytic* or antifibrinolytic* or antifibrinolysin* or anti-fibrinolysin* or antiplasmin* or anti-plasmin* or ((plasmin or fibrinolysis) adj3 inhibitor*)).ab,ti.

3. exp Tranexamic Acid/

4. (tranexamic or Cyclohexanecarboxylic Acid* or Methylamine* or amcha or trans-4-aminomethyl-cyclohexanecarboxylic acid* or t-amcha or amca or kabi 2161 or transamin* or exacyl or amchafibrin or anvito or spotof or cyklokapron or ugurol oramino methylcyclohexane carboxylate or aminomethylcyclohexanecarbonic acid or aminomethylcyclohexanecarboxylic acid or AMCHA or amchafibrin or amikapron or aminomethyl cyclohexane carboxylic acid or aminomethyl cyclohexanecarboxylic acid or aminomethylcyclohexane carbonic acid or aminomethylcyclohexane carboxylic acid or aminomethylcyclohexanecarbonic acid or aminomethylcyclohexanecarboxylic acid or aminomethylcyclohexanocarboxylic acid or aminomethylcyclohexanoic acid or amstat or anvito or cl?65336 or cl65336 or cyclocapron or cyclokapron or cyklocapron or exacyl or frenolyse or hexacapron or hexakapron or tranex or TXA).ab,ti.

5. 1 or 2 or 3 or 4

6. exp Administration, Topical/

7. (topical* or (local* adj3 appl*) or irrigat* or spray*).ti,ab.

8. 6 or 7

9. 5 and 8

10. exp Spine/

11. exp Spinal Fusion/

12. 10 or 11

13. 9 and 12

16. randomized controlled trial/

17. single-blind procedure/

18. random*.mp.

19. placebo*.mp.

20. (double* adj blind*).mp.

21. (singl* adj blind*).mp.

22. exp Cohort Studies/

23. (cohort* or prospective* or retrospective*).mp.

24. 16 or 17 or 18 or 19 or 20 or 21 or 22 or 23

25. exp animals/ not humans.sh.

26. 24 not 25

27. 13 and 26

## Abbreviations

ivTXA: Intravenous tranexamic acid administration
tTXA: Topical use of TXA
RevMan: Review Manager
CI: Confidence interval
RCTs: Randomized controlled trials
qi-RCTs: Quasi-randomized trials
HBL: Hidden blood losses
MD: Mean difference
IQR: Interquartile range
LOH: Length of hospital stay
PAP: Plasmin/α_2_-antiplasmin
ERAS: Enhanced recovery after surgery
